# Autoimmunity and inborn errors of immunity: a complex coexistence

**DOI:** 10.3389/fimmu.2026.1787739

**Published:** 2026-03-11

**Authors:** Ahamada Elamine, Ibtihal Benhsaien, Fatima Ailal, Mohammed Hbibi, Mohammed Fahi, Ahmed Aziz Bousfiha, Jalila EL Bakkouri

**Affiliations:** 1 Laboratory of Clinical Immunology, Infection and Autoimmunity (LICIA), Faculty of Medicine and Pharmacy, Hassan II University, Casablanca, Morocco; 2 Department of Pediatric Infectious Diseases and Clinical Immunology, Mother-Child Hospital Abderrahim El Harouchi, Ibn Rochd University Hospital Center, Casablanca, Morocco; 3 Immuno-serology Laboratory, Ibn Rochd University Hospital Center, Casablanca, Morocco; 4 Immunopathology-Immunotherapy-Immunomonitoring Laboratory, Faculty of Medicine, Mohammed VI University of Health Sciences (UM6SS), Casablanca, Morocco

**Keywords:** autoimmunity, diagnostic approach, immune dysregulation, immune tolerance defects, inborn errors of immunity

## Abstract

Inborn errors of immunity (IEI) and autoimmune diseases represent two major consequences of immune system dysregulation. Although recurrent infections are a hallmark of IEI, autoimmune manifestations are also highly prevalent in affected patients. Nevertheless, identifying an underlying IEI in individuals presenting primarily with autoimmune disorders remains challenging, as these manifestations can be highly heterogeneous. Many pediatricians and specialists managing autoimmune conditions do not systematically consider an early immunological evaluation, often underestimating the likelihood of an underlying immunodeficiency. As a result, the diagnosis of a genetic immunodeficiency is frequently delayed. The pathogenesis of autoimmune manifestations in IEI is complex and largely related to defects in central and peripheral immune tolerance, leading to the persistence of autoreactive T and B lymphocytes. Consistently, pathogenic variants in genes that play a critical role in the establishment and maintenance of immune tolerance have been identified in patients with IEI.

Through this narrative review, we aim to raise awareness among clinicians and laboratory specialists about the close interplay between autoimmunity and IEI, and to emphasize that autoimmune manifestations may represent an early warning sign of an underlying immunodeficiency. We also highlight key elements to guide the diagnostic approach to autoimmunity in this clinical context.

## 1 Introduction

Inborn errors of immunity (IEI), formerly referred to as primary immunodeficiencies (PID), comprise a heterogeneous group of more than 500 inherited disorders affecting the development and function of the immune system (1–3). Over the past decade, the relationship between IEI and autoimmunity has been extensively investigated. Patients with IEI may develop varying degrees of immune dysregulation, resulting in a broad clinical spectrum characterized by both infectious susceptibility and autoimmune manifestations (4).

This complex interplay between IEI and autoimmunity has been well illustrated by studies focusing on specific disease entities, such as Evans syndrome (ES). Recent evidence suggests that ES frequently represents a manifestation of an underlying inborn error of immunity and may therefore require tailored diagnostic and therapeutic approaches. For instance, an Italian cohort study including 40 patients with ES demonstrated that genetic analysis identified pathogenic variants associated with primary immune regulatory disorders (PIRD) and IEI in 45% of cases (5).

Autoimmune manifestations are common in patients with IEI and may constitute the initial clinical presentation of an underlying immunodeficiency (6). Data from the French Primary Immunodeficiency Registry showed that 26.2% of patients with IEI developed one or more autoimmune or inflammatory complications (7). Similarly, the Kuwaiti Primary Immunodeficiency Registry reported a prevalence of autoimmunity of 19% among affected individuals (9). In Morocco, among 769 registered patients, 108 (14%) presented with at least one autoimmune manifestation (8). In patients in whom autoimmunity coexists with IEI, appropriate and timely management is crucial, as this combination is associated with an increased risk of mortality (9). This narrative review aims to synthesize current knowledge on the coexistence of autoimmunity and IEI. We review the main autoimmune manifestations observed in patients with IEI and discuss the underlying pathophysiological mechanisms in selected forms of IEI that are strongly associated with autoimmunity. Finally, we address diagnostic strategies for evaluating autoimmunity in patients with IEI, as well as the assessment of IEI in patients presenting with autoimmune disease.

### 1.1 Search strategy and selection criteria

To provide a comprehensive overview of the coexistence between autoimmunity and inborn errors of immunity, we conducted a narrative review of the literature. The bibliographic search was performed using PubMed, Web of Science, and Google Scholar. The search focused on peer-reviewed articles consulted during the period 2024–2025, with particular emphasis on studies from the past five years in order to capture recent advances in the field. The search strategy included combinations of the keywords “inborn errors of immunity,” “autoimmunity,” “immune dysregulation,” as well as the names of relevant genes and clinical entities. Systematic reviews, narrative reviews, observational cohort studies, and prospective studies were included. Conference abstracts lacking complete peer-reviewed data were excluded.

## 2 Epidemiology of autoimmunity in inborn errors of immunity

This section addresses the epidemiological data on the association between autoimmunity and IEI, with a particular focus on the reported prevalence of this coexistence across different cohorts and geographic regions. The available evidence is derived primarily from national and international cohort studies, reflecting the heterogeneity of the studied populations and the methodologies employed.

Although the association between autoimmunity and IEI has been investigated in multiple studies, the global prevalence of this coexistence remains poorly defined. Published data on the frequency of autoimmune manifestations in IEI are often limited to specific IEI entities, selected subgroups, or particular autoimmune phenotypes (10). Alain Fischer et al. reported a prevalence of autoimmunity of 26.2% among 2,183 patients with IEI included in a national cohort (7). Similarly, a study conducted in Slovenia involving 235 patients with IEI identified autoimmune manifestations in 22% of cases (10).

Below, we summarize a series of studies from different countries highlighting the substantial variability in the reported frequency of autoimmune manifestations associated with IEI (**Table 1**).

**Table 1:** Prevalence of autoimmunity across different IEI registries.

Country, publication date'	Number of IEI cases	Frequency of autoimmunity	References
Morocco, 2025	769	14%	(8)
France, 2017	2183	26,2%	(7)
Slovenia, 2016	235	22%	(10)
Kuwaït, 2020	286	19,9%	(9)
Iran, 2021	461	20%	(11)
Turkey, 2020	822	10,1%	(12)
Mexico, 2021	40	7,5%	(13)
Tunisia, 2014	710	4,2%	(14)

These epidemiological data indicate that autoimmune manifestations are relatively frequent among patients with IEI. The substantial variability in reported prevalence highlights the heterogeneity of IEI, methodological differences between studies, and the likely underestimation of this association. Collectively, these findings underscore the need for large-scale multicenter studies using standardized criteria to better define the true burden of autoimmunity in IEI.

## 3 Clinical spectrum of autoimmune manifestations in inborn errors of immunity

Among the autoimmune manifestations most frequently associated with IEI are autoimmune cytopenias, organ-specific autoimmune diseases, and selected rheumatologic conditions. Below, we highlight representative examples within each of these categories.

### 3.1 Autoimmune cytopenias

Autoimmune cytopenias represent the most common autoimmune complications observed in patients with IEI (15). Among these, immune thrombocytopenia (ITP) is the most frequently reported, followed by autoimmune hemolytic anemia (AIHA). The prevalence of autoimmune cytopenias varies depending on the specific type of IEI. In patients with common variable immunodeficiency (CVID), approximately 25% develop autoimmune cytopenias, including ITP in 15%, AIHA in 5%, and ES defined by the coexistence of ITP and AIHA in about 4% of cases. Notably, in nearly 75% of patients, autoimmune cytopenias precede the onset of recurrent infections related to humoral immunodeficiency (16, 17).

Given that Evans syndrome is reported in up to 50% of patients with autoimmune lymphoproliferative syndrome (ALPS), the diagnosis of ALPS should be systematically considered in patients presenting with ES (17). In addition, autoimmune neutropenia has been described in patients with CVID as well as in other forms of IEI (6).

### 3.2 Organ-specific autoimmune diseases

A wide spectrum of organ-specific autoimmune manifestations has been reported in patients with IEI (15, 18, 19). These include, but are not limited to, the following conditions:

#### 3.2.1 Autoimmune enteropathy and inflammatory colitis

These manifestations are particularly prevalent in immune dysregulation, polyendocrinopathy, enteropathy, X-linked (IPEX) syndrome, and are observed in up to 10% of patients with CVID. Similar gastrointestinal involvement has also been described in Lipopolysaccharide-Responsive Beige-like Anchor protein (LRBA) and Cytotoxic T-Lymphocyte Antigen-4 (CTLA-4) deficiencies affecting up to 80% of patients as well as in gain-of-function mutations of Phosphoinositide-3-kinase catalytic subunit delta (PIK3CD), Omenn syndrome, and Wiskott-Aldrich syndrome (WAS).

#### 3.2.2 Celiac-like disease without autoantibodies

A celiac disease-like enteropathy lacking classical autoantibodies has been reported in approximately 5–10% of patients with CVID.

#### 3.2.3 Autoimmune hepatitis and glomerulonephritis

These manifestations have been associated with hyper-IgM syndromes and CVID.

#### 3.2.4 Autoimmune endocrinopathies

Autoimmune thyroiditis and type 1 diabetes mellitus are hallmark features of autoimmune polyendocrinopathy-candidiasis-ectodermal dystrophy (APECED), but are also observed in IPEX syndrome and CVID. In CTLA-4 deficiency, autoimmune thyroiditis occurs in nearly one-third of patients, while type 1 diabetes mellitus is reported in approximately 15% of cases.

#### 3.2.5 Less frequent organ involvement

Rare autoimmune manifestations include uveitis and inflammatory or granulomatous central nervous system disorders (20).

### 3.3 Inflammatory articular manifestations

Inflammatory joint manifestations are frequently observed in patients with IEI, particularly in those with CVID, where they affect approximately 15% of patients, and in hyper-IgM syndromes (15). These manifestations may correspond to true inflammatory rheumatic diseases, such as juvenile idiopathic arthritis or rheumatoid arthritis. However, joint involvement may also result from infectious etiologies related to the underlying immunodeficiency, including viral replication (Epstein–Barr virus or cytomegalovirus), parvovirus B19 infection, or *Mycoplasma* infection. In addition, certain systemic autoimmune diseases, such as systemic lupus erythematosus (SLE), Sjögren’s syndrome, or sarcoidosis, may present with persistent and profound T-cell lymphopenia or coexist with genuine humoral immunodeficiencies, particularly CVID (21).

In summary, autoimmune cytopenias are the most frequent autoimmune manifestations in patients with inborn errors of immunity and may precede infectious complications, serving as an early clinical clue to the underlying disorder. Organ-specific autoimmune diseases, particularly gastrointestinal and endocrine involvement, are also common, especially in immune dysregulation syndromes. These findings emphasize the importance of considering IEI in patients presenting with early-onset, severe, or multisystem autoimmunity.

## 4 Shared mechanisms between autoimmunity and inborn errors of immunity

**Figure 1:**
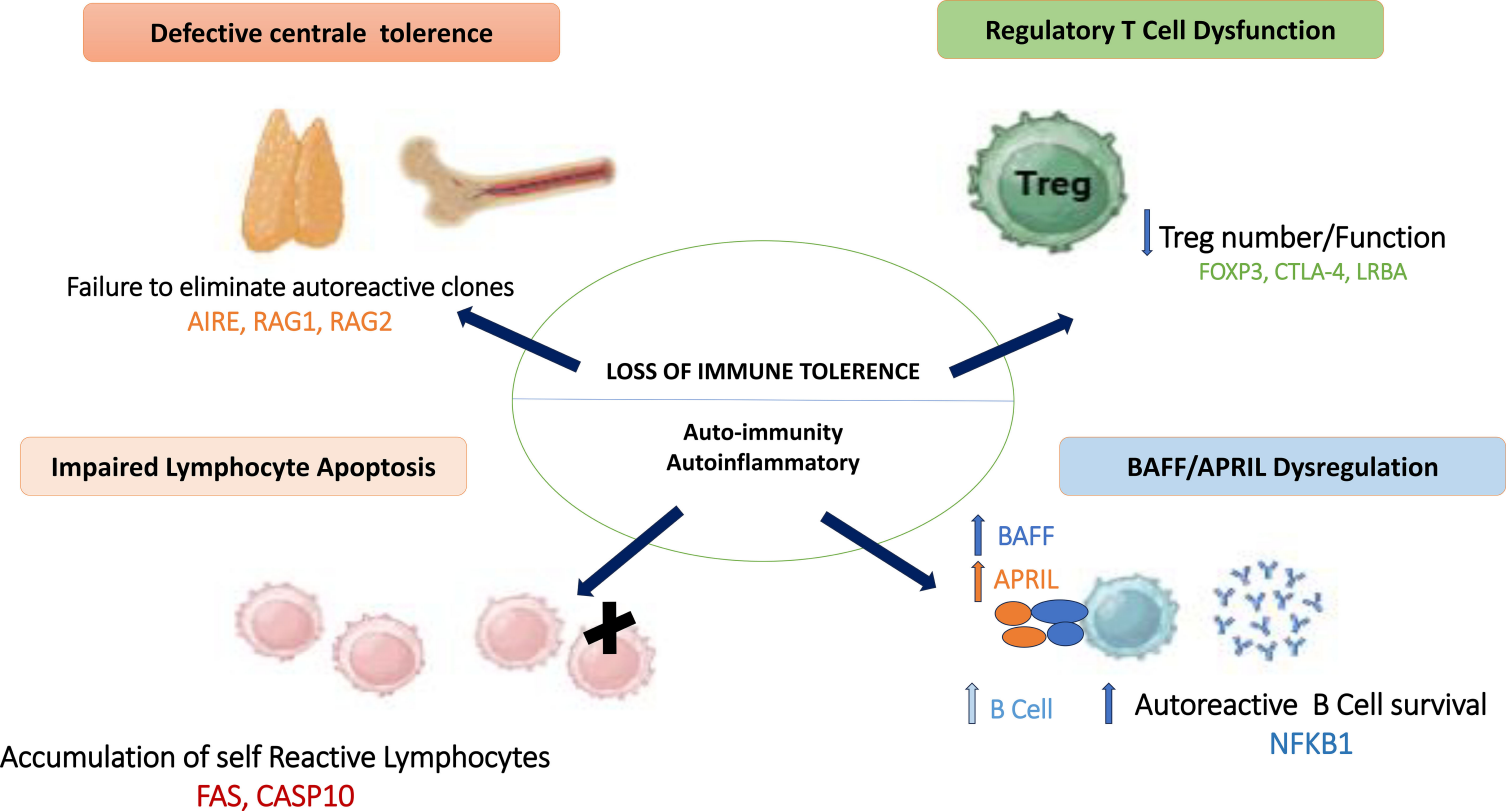
Shared mechanisms of immune tolerance breakdown in inborn errors of immunity. Schematic overview of the shared immunopathological mechanisms involved in autoimmunity associated with IEI, including defective central tolerance, impaired regulatory T cells, abnormal lymphocyte apoptosis, and dysregulation of the BAFF/APRIL axis, leading to loss of immune tolerance and autoimmune manifestations. AIRE, AutoImmune Regulator; RAG1, Recombination Activating Gene 1; RAG2, Recombination Activating Gene 2; FOXP3, Forkhead Box P3; CTLA4, Cytotoxic T-Lymphocyte–Associated Protein 4; LRBA, Lipopolysaccharide-Responsive Beige-Like Anchor Protein; NFKB1, Nuclear Factor Kappa B Subunit 1; BAFF, B-cell Activating Factor; APRIL, A Proliferation-Inducing Ligand; FAS, Fas Cell Surface Death Receptor; CASP10, Caspase 10.

Inborn errors of immunity and autoimmunity were initially considered as opposing extremes of immune function. However, the high prevalence of autoimmune diseases in patients with IEI has strengthened the hypothesis of shared underlying mechanisms (22). Defects in central and peripheral immune tolerance, along with the persistence of autoreactive T and B lymphocytes, represent key features common to both conditions (**Figure 1**) (23). Furthermore, pathogenic variants in genes critical for the development and maintenance of immune tolerance have been identified in patients with IEI (24). Below, we discuss specific types of IEI that are consistently or strongly associated with autoimmune manifestations (25).

### 4.1 Common variable immunodeficiency

Worldwide, CVID remains the second most frequent primary immunodeficiency. It may present in either autosomal dominant or autosomal recessive forms, with approximately 15% of cases being recessive. According to the International Consensus (ICON), CVID is characterized by low serum levels of IgG and IgA, impaired IgM production, and absent or weak responses to specific antibodies. The pathogenesis of CVID has been extensively studied. Clinically, four major immune defects are frequently observed: hypogammaglobulinemia, impaired T-cell activation and proliferation, dendritic cell dysfunction, and cytokine deficiencies (26).

Loss of T-cell function and impaired proliferation leads to reduced circulating CD4+ T cells and regulatory T cells, predisposing patients to autoimmunity or chronic inflammation (27). Approximately 20% of CVID patients develop autoimmune manifestations, with autoimmune cytopenias such as AIHA and ITP being the most frequent (28–30). B cells in CVID are often present in normal or elevated numbers, but display abnormal maturation and reduced immunoglobulin production (31). Recent studies suggest that defects in class-switch recombination and impaired somatic hypermutation may contribute to the persistence of autoreactive clones, thereby promoting autoimmunity (32, 33). Moreover, CVID patients exhibit elevated levels of proliferation-inducing ligand (APRIL) and B cell–activating factor (BAFF), which have been associated with systemic autoimmune diseases such as rheumatoid arthritis (RA) and SLE (34–36). These patients also show reduced levels of immunomodulatory cytokines including IL-2, IL-4, and IL-5 as well as decreased expression of forkhead box P3 (FoxP3) in regulatory T cells (Tregs). Collectively, these findings suggest that CVID is associated with defects in both central and peripheral immune tolerance, contributing to the development and maintenance of autoimmune phenomena (37).

### 4.2 Autoimmune polyendocrinopathy–candidiasis–ectodermal dystrophy

APECED is caused by mutations in the autoimmune regulator (AIRE) gene and is inherited in an autosomal recessive manner (24). Clinically, the syndrome is characterized by adrenal insufficiency, hypoparathyroidism, and chronic mucocutaneous candidiasis. Diagnosis is established when at least two of these three classical features are present (38).

Patients with APECED may also develop systemic autoimmune manifestations, including vitiligo, hypothyroidism, and arthritis (39). Genetic studies have suggested associations between specific AIRE polymorphisms and the risk of autoimmune diseases. Garcia-Lozano et al. reported that the rs878081c variant was associated with the development of RA (40). Similarly, Berczi et al. identified rs2075876 and rs760426 as polymorphisms linked to an increased risk of RA, particularly in Asian populations (41). Functional studies have demonstrated that patients with APECED exhibit T-cell regulatory defects, including reduced FoxP3 expression and impaired activation of peripheral Tregs (42). The autoimmune manifestations observed in APECED are therefore likely the consequence of combined defects in central and peripheral immune tolerance.

### 4.3 Immune dysregulation, polyendocrinopathy, enteropathy, X-linked syndrome

IPEX is an X-linked recessive syndrome caused by mutations in the FoxP3 gene. FoxP3 is essential for the development, transcriptional regulation, and function of Tregs and is also implicated in the regulation of IgA and IgE production by B lymphocytes (43). The clinical presentation of IPEX is highly variable, but the classical triad includes polyendocrinopathy, enteropathy, and eczematous dermatitis (22). Gastrointestinal symptoms are the most common and typically manifest around six months of age. IPEX usually presents early in life and evolves over time with the emergence of new clinical signs (44).

Endocrine manifestations are dominated by type 1 diabetes mellitus and autoimmune thyroid disease (45). Hwang et al. reported that FoxP3 mutations may underlie the development of type 1 diabetes (46). Additional associated features include hypothyroidism, AIHA, ITP, eczema, and atopy in some patients (44, 47, 48).

The mechanisms linking autoimmunity to IPEX have been increasingly studied. Chene et al. demonstrated elevated GATA binding protein 3 (GATA-3) expression in T cells from the intestines an d kidneys of IPEX patients, which decreased after immunomodulatory treatment (49). Patients also exhibit an expansion of Th17 cells, accompanied by elevated levels of IL-17, IL-6, and IL-23. Furthermore, due to defective Treg responses, mature naïve B cells in IPEX patients produce autoreactive antibodies (50). These findings highlight the critical role of Tregs in the development of autoimmunity in primary immunodeficiencies and suggest that Treg-targeted therapies may represent a promising approach for these patients.

### 4.4 Autoimmune lymphoproliferative syndrome

Autoimmune lymphoproliferative syndrome is primarily caused by mutations in the FAS gene (approximately 70% of cases), and less frequently in CASPASE 10 and FASL. This relatively rare syndrome is usually inherited in an autosomal dominant manner. The interaction between FAS, FASL, and CASPASE 8 and 10 initiates a signaling cascade that leads to proteolysis, DNA fragmentation, and apoptosis (51). Mutations in these genes result in defective lymphocyte apoptosis, disrupting immune homeostasis and promoting the accumulation of autoreactive lymphocytes in secondary lymphoid organs (52, 53).

Advances in genetics and genome sequencing have identified additional candidate genes, including KRAS, NRAS, CTLA4, LRBA, MAGT1, STAT3, and TNFAIP3 (54). Clinically, ALPS exhibits heterogeneous manifestations, including various hematological abnormalities. Lymphoproliferation is the most common feature, which may present as lymphadenopathy, hepatomegaly, and/or splenomegaly, often persisting throughout life (55). Autoimmune manifestations represent the second most frequent clinical feature, with a predominance of autoimmune cytopenias, including AIHA, ITP, and autoimmune neutropenia (55–57).

The FAS gene plays a central role in lymphocyte regulation and peripheral tolerance, contributing both to immune defense and prevention of autoimmunity. Mutations affecting apoptotic pathway receptors disrupt immune homeostasis, leading to the accumulation of autoreactive lymphocytes and predisposing patients to autoimmune manifestations (55). This mechanism is considered the principal explanation for the autoimmunity observed in ALPS patients.

### 4.5 Wiskott–Aldrich syndrome

Wiskott–Aldrich syndrome is classically characterized by the triad of thrombocytopenia, eczema, and recurrent infections, which may be caused by viral, bacterial, or encapsulated pathogens (58). This rare disorder follows an X-linked mode of inheritance and is caused by mutations in the WAS gene encoding the WAS protein (WASp), which is expressed in the cytoplasm of hematopoietic cells. WASp plays a critical role in signal transduction from the cell surface to the actin cytoskeleton and is essential for immune cell function (59). Patients with WAS exhibit defects in both humoral and cellular immunity (59).

The risk of autoimmune disease is markedly increased in patients with WAS. A French cohort study assessing the frequency of autoimmune manifestations in WAS patients reported AIHA as the most common manifestation (36%), followed by arthritis (29%), autoimmune neutropenia (25%), and vasculitis (22%) (60–62). Experimental studies using antigen-induced arthritis models in WASp-deficient mice have demonstrated a reduction in regulatory T and B cells, along with an expansion of Th17 cells, providing mechanistic insight into the heightened susceptibility to autoimmunity in this condition (63).

Autoimmune manifestations represent a major and increasingly recognized component of the clinical spectrum of IEI. Far from being exceptional, autoimmunity often constitutes a defining feature of immune dysregulation and, in many cases, may precede the onset of recurrent infections, thereby masking the underlying immunodeficiency and delaying diagnosis. The data reviewed herein highlight that autoimmunity is not restricted to a limited subset of IEI but spans a wide range of disorders, including CVID, APECED, IPEX, ALPS, hyper-IgM syndromes, and Wiskott–Aldrich syndrome. Despite their genetic and clinical heterogeneity, these disorders share common mechanisms of immune dysregulation, such as defects in central and peripheral tolerance, impaired regulatory T-cell function, abnormal lymphocyte apoptosis, and imbalances in effector T-cell subsets. These alterations promote the persistence of autoreactive lymphocytes and the development of autoimmune disease. From a clinical perspective, recognizing autoimmunity as a potential warning sign of IEI is crucial. The presence of autoimmune cytopenias or early-onset, atypical, or refractory autoimmune diseases should prompt immunological and genetic evaluation. An integrated diagnostic approach enables tailored management and improves outcomes in patients with IEI-associated autoimmunity.

## 5 Autoimmunity and inborn errors of immunity: a cross-diagnostic approach

### 5.1 When and how should IEI be diagnosed in patients with autoimmune manifestations?

Autoimmune manifestations may represent early warning signs of IEI and should prompt clinicians to consider an underlying immunodeficiency in specific clinical contexts. These include the onset of autoimmunity at a young age, the presence of polyautoimmunity, autoimmune cytopenias, autoimmunity occurring after infections, and a family history of autoimmune diseases. Together, these features constitute red flags strongly suggesting an underlying (64).

**Figure 2:**
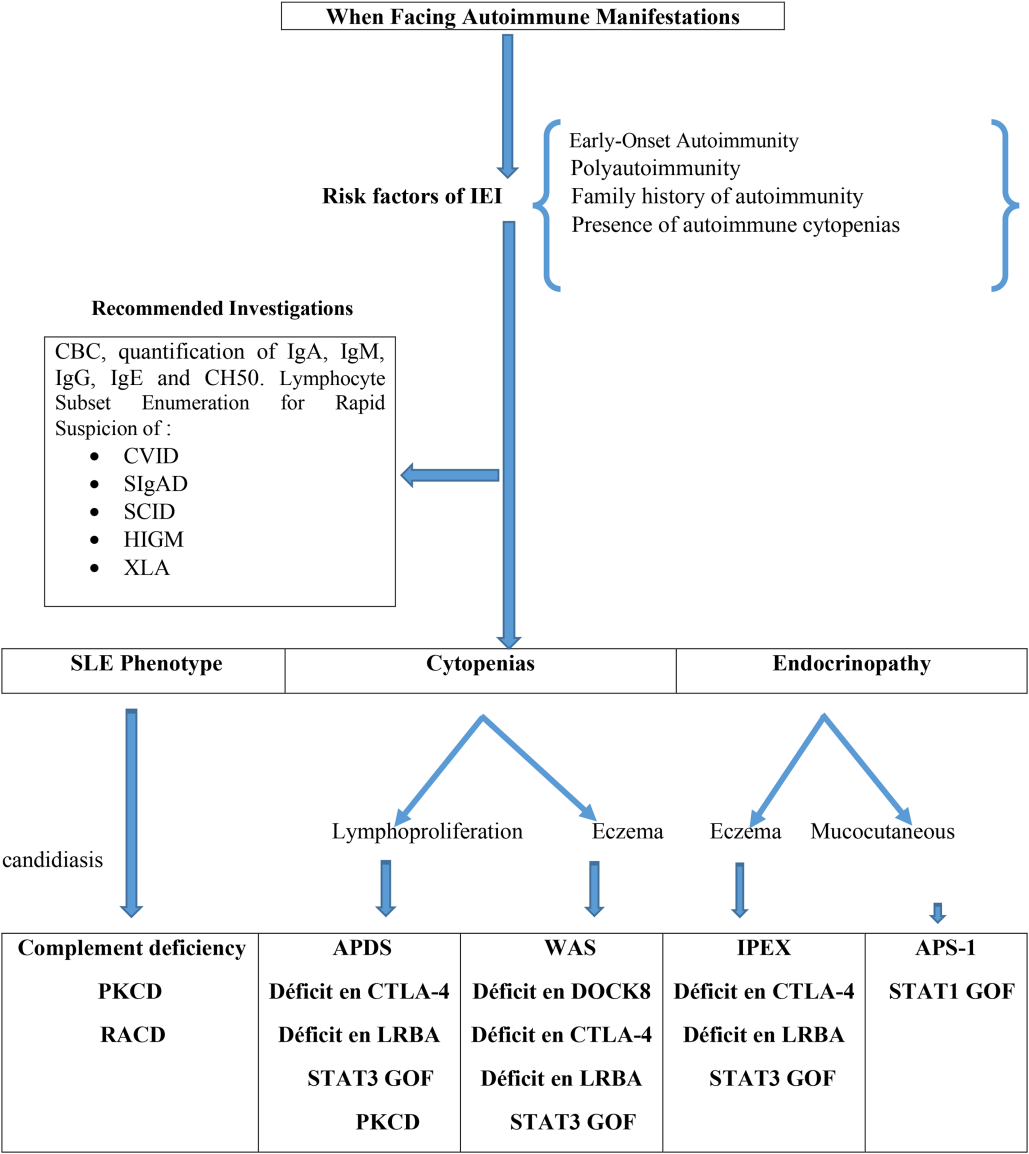
Diagnostic approach for IEI in patients presenting with autoimmunity (27). APDS, Activated phosphoinositide 3-kinase d syndrome; APS-1, Autoimmune polyendocrine syndrome 1; CTLA-4, Cytotoxic lymphocyte T antigen 4; CVID, Common variable immunodeficiency; HIGM, Hyper-IgM syndrome; LRBA, LPS-responsive beige-like anchor protein; PKCD, protein kinase C δ deficiency; RALD, Ras-associated leukoproliferative disorder; SCID, severe combined immunodeficiency; sIgAD, Selective IgA deficiency; SLE, systemic lupus erythematosus; STAT, Signal Transducers and Activator of Transcription; WAS, Wiskott-Aldrich syndrome; XLA, X-linked agammaglobulinemia.

A structured diagnostic approach in patients with autoimmune diseases should begin with a thorough assessment of clinical history. This step is essential to identify infectious episodes, evaluate their severity and frequency, and detect clinical features suggestive of IEI. Key elements include failure to thrive, recurrent or severe infections, the need for hospitalization due to infections, prolonged or repeated antibiotic use, and infections caused by unusual or opportunistic pathogens. In addition, a baseline immunological evaluation should be systematically performed in all patients with autoimmune manifestations, including measurement of serum immunoglobulin levels and analysis of lymphocyte subsets (**Figure 2**).

The extent of further investigations should be guided by the specific autoimmune phenotype. Particular attention should be given to patients presenting with autoimmune cytopenias. These manifestations are not only common features of a wide range of IEI but may also represent the initial presentation of systemic connective tissue diseases, such as SLE (65). Consequently, the diagnostic work-up in these patients should combine an assessment of immune function with targeted screening for relevant autoantibody subsets, including antinuclear and antithyroid antibodies (66). Notably, patients with IEI have a 120-fold increased risk of developing autoimmune cytopenias, with a particularly high incidence of AIHA (7). Individuals presenting with multiple cytopenias warrant special consideration, as studies in ES have shown that nearly half of affected children harbor pathogenic variants associated with IEI. This subgroup is also characterized by a high prevalence of systemic autoimmune diseases (67, 68).

IEI should also be suspected in patients with autoimmune endocrine disorders, particularly when disease onset occurs earlier than expected, when two or more endocrine disorders coexist, or when additional features suggestive of IEI are present. In this context, clinical signs such as eczema, elevated serum IgE levels, and peripheral eosinophilia should raise suspicion for disorders involving Treg dysfunction (69). Moreover, in patients presenting with SLE-like manifestations, measurement of serum complement fractions is recommended as part of the diagnostic evaluation (70).

Finally, establishing a definitive diagnosis requires more advanced investigations tailored to the suspected clinical phenotype. These include assessment of vaccine-induced immune responses (particularly relevant for the clinical diagnosis of CVID), comprehensive immunophenotyping of lymphocyte subsets including memory B and T cells functional immune assays, and, when indicated, cytogenetic and genetic testing.

### 5.2 When and how should autoimmunity be diagnosed in children with IEI?

Regular monitoring for autoimmune manifestations is essential in patients with IEI, particularly in children carrying genetic defects strongly associated with autoimmunity, such as mutations in CTLA4, LRBA, or PI3K. Increasing attention has been directed toward the identification of immunological predictors of autoimmunity in this population. However, to date, no single immunological marker has demonstrated strong and reliable predictive value for the development of autoimmune disease. Nevertheless, data derived from cohorts of patients with primary antibody deficiencies and combined immunodeficiencies (CID) have highlighted several promising candidate biomarkers.

Large-scale studies in patients with CVID and autoimmune cytopenias have shown that elevated serum immunoglobulin levels, increased frequencies of CD19^+^ B cells, and expanded CD4^+^ effector T-cell populations are associated with a reduction in naïve T cells. The absence or reduction of memory B cells has been correlated with autoimmune cytopenias, systemic autoimmune diseases, splenomegaly, granulomatous disease, and lymphadenopathy (71, 72). In addition, patients with CVID and autoimmunity exhibit decreased numbers of Treg (73), along with an expansion of CD21^low^ B cells. These CD21l^ow^ B cells display enhanced IgM production following stimulation with CD40L, IL-2, and IL-10 (74, 75). Suggesting that elevated IgM levels may serve as a marker of autoimmunity and reflect a potential pathogenic mechanism. Furthermore, these patients demonstrate reduced proportions of naïve CD4^+^ and CD8^+^ T cells with a concomitant increase in differentiated T-cell subsets, a pattern that may contribute to the breakdown of immune tolerance (76).

In the context of combined immunodeficiency, a recent study by Montin et al. in patients with 22q11.2 deletion syndrome identified specific immunological features associated with the development of hematological autoimmunity. These included reduced numbers of naïve T cells, decreased recent thymic emigrants (RTEs), and an increased proportion of naïve B cells. Importantly, these abnormalities were detectable well before the onset of clinically overt autoimmunity (77). Moreover, the degree of T-cell lymphopenia has been proposed as a contributing factor to autoimmune manifestations in this condition (78).

Collectively, these findings underscore the importance of detailed and longitudinal immunological assessment in the follow-up of patients with IEI. Such an approach may facilitate early detection of autoimmune complications and provide valuable insights into the immunopathological mechanisms underlying autoimmunity in this vulnerable population.

## 6 Conclusion

It is widely recognized that autoimmunity and IEI frequently coexist, with autoimmune manifestations often representing the earliest clinical signs of an underlying IEI. However, early-onset autoimmunity, the presence of polyautoimmunity, infection-associated autoimmune phenomena, and a family history of autoimmune disease should be regarded as warning signals prompting evaluation for an underlying IEI. Comprehensive immunological assessment, together with timely genetic testing, is essential to establish an accurate diagnosis and to optimize patient management. Furthermore, advances in the understanding of the pathophysiological mechanisms underlying immune dysregulation have opened new therapeutic perspectives, including targeted therapies and immunomodulatory approaches, enabling more personalized and effective long-term care for patients with IEI-associated autoimmunity.
